# Perceptions and experiences of COVID-19 vaccines’ side effects among healthcare workers at an Egyptian University Hospital: a cross-sectional study

**DOI:** 10.1186/s41182-022-00427-2

**Published:** 2022-05-30

**Authors:** Hisham Ahmed Orebi, Hesham Elsayed Emara, Abdallah Ahmoud Alhindi, Mohamed Reda Shahin, Arwa Hassan Hegazy, Ibrahim Ali Kabbash, Shimaa M. Saied

**Affiliations:** 1 Faculty of Medicine, Tanta University, Tanta, Egypt; 2 Public Health and Community Medicine Department, Faculty of Medicine, Tanta University, Tanta, Egypt

**Keywords:** COVID-19, Vaccine, Side effects, Healthcare workers, Egypt

## Abstract

**Background:**

A safe and effective vaccine is the ultimate key to mitigating the COVID-19 pandemic. Vaccine acceptance is influenced by various factors, including perceptions about the vaccine’s safety and side effects. The side effects vary depending on the type of the vaccine, but they are mainly mild, local, temporary, and self-limiting.

**Methods:**

A cross-sectional study was carried out at Tanta University Hospitals, including 1246 healthcare workers who received either the first or the second dose of the COVID-19 vaccine, selected via a systematic random sampling technique using a self-administered structured validated questionnaire for data collection from November 2021 to January 2022. Qualitative data were presented as frequencies and percentages and analyzed using Chi-square and Fisher’s exact tests.

**Results:**

The prevalence of one or more side effects was 91.3%. Among participants, about two-thirds believed in vaccine safety and its necessity (65.4% and 63.6%, respectively). Significantly more participants (46.9%) were concerned about AstraZeneca thrombotic complications than other vaccine types. The top five side effects reported by participants were injection site pain (64.8%), sense of fatigue (57.1%), headache (49.9%), muscle pain (48.7%), and fever (46.5). Most of the side effects were significantly higher among participants vaccinated with AstraZeneca. Side effects impacted work capacity of 23.4%, which was significantly higher among participants who received AstraZeneca (33.6%).

**Conclusion:**

Participants had a good level of belief in vaccination safety and necessity. Healthcare workers who got the AstraZeneca vaccination reported more adverse effects than other vaccines. Injection site pain, fatigue, headache, muscle pains, and fever were the most frequently reported side effects. More research on vaccination safety is needed to understand the long-term adverse effects of vaccinations better, improve the public trust, and accelerate vaccine adoption.

## Background

Severe acute respiratory syndrome coronavirus 2 (SARS-CoV-2), the virus responsible for coronavirus disease (COVID-19), is a global crisis that has hit worldwide since its emergence in December 2019 [1]. Infected individuals may have mild symptoms, such as fever, cough, myalgia, and fatigue, or may suffer from pneumonia, multiple organ failure, and die in some cases [2]. Healthcare workers (HCWs) are at risk of contracting COVID-19 [3]. Besides the increased mortality risk, various studies have revealed that COVID-19 hurts HCWs’ psychological and well-being status [4]. A systematic review reported the prevalence of COVID-19-positive HCWs throughout the early months of the COVID-19 pandemic as 51.7%, and a multicenter study from Egypt demonstrated the seroprevalence of anti-SARS-CoV-2 antibodies as 46.3% [5, 6]. We are experiencing a rapid spread due to SARS-CoV-2 because of its continuous mutations and quickly spreading infection [7]. Thus, a safe and effective vaccine is the ultimate key in mitigating the COVID-19 pandemic [8].

The start of the COVID-19 vaccine rollout in December 2020 was a landmark in the history of the fight against this pandemic; thus, it was recommended that the pandemic history be divided into pre-vaccination and post-vaccination periods [9]. Vaccine hesitancy is a major public health concern, fueled by rumors and misinformation about vaccine effectiveness and safety [10]. Vaccine acceptance can be influenced by various factors, including knowledge of the vaccine, perceptions about its adverse effects, attitude toward vaccination, perceived vulnerability to illness, social impacts, trust in the healthcare profession, and increased vaccine information [11]. A large-scale multinational study concluded that the highest rates of vaccination hesitancy were found among HCWs from the Arab world’s western areas (Egypt, Morocco, Tunisia, and Algeria). Concerns about adverse effects were the most often reported reason for hesitancy [12]. The different types of the available vaccines are mRNA vaccines (Pfizer BioNTech, Moderna, and Johnson & Johnson), viral vector vaccines (AstraZeneca, Sputnik V), and inactivated vaccines (Sinopharm, Sinovac, and COVAXIN) are among the COVID-19 vaccines now widely available for usage worldwide [13]. Understanding the potential side effects of vaccination is essential for all groups engaged in the process, including the individual who receives the vaccine, caregivers, and healthcare providers [14]. Maximizing the COVID-19 vaccination rate among healthcare workers (HCWs) is an evidence-based, reasonable approach to public health priorities [15]. Healthcare workers’ awareness of the significance of vaccination programs influences their attitudes towards public health preventive measures, especially when considering a long-term anti-COVID-19 strategy requiring future doses of booster vaccinations [16]. Furthermore, healthcare practitioners serve as the general public’s guide and trusted source of vaccination information. They can guard against false and confusing information. As a result, their attitude will influence their own and others’ health [17]. Previous research on the side effects of the COVID-19 vaccine found mild-to-moderate side effects, with the severity of adverse effects varying depending on the type of COVID-19 vaccine used [18]. Most of the reported side effects were moderate, such as fever, headache, local pain at the injection site, and muscle pain [19]. Since the development and availability of COVID-19 vaccinations, governments worldwide have actively worked to implement effective mass vaccination programs [20].

When the COVID-19 vaccine became available in Egypt, the Egyptian Ministry of Health (MOH) prioritized healthcare workers. They were the highest risk population for being infected with the new virus. The earliest vaccinated HCWs were given the Oxford–AstraZeneca COVID-19 vaccine until the all-available doses ran out, then they received the Sinopharm and Sinovac vaccines [17, 21]. According to WHO, until May 2022, Egypt had 513,790 confirmed COVID-19 cases, including 24,641 deaths, and a total of 82,017,392 vaccination doses were delivered [22]. A study of COVID-19 vaccines` side effects among the Egyptian population concluded that coronavirus vaccinations were well-tolerated and safe, with most post-vaccine adverse effects being mild to moderate [23].

Few studies [21, 23] were carried out to increase knowledge about the COVID-19 vaccine’s adverse effects by enquiring about and assessing self-reported side effects across various demographic and medical characteristics. Therefore, the primary objective of this study was to assess the perceptions and the frequency of the experienced side effects of the early-vaccinated Tanta University Hospitals’ HCWs regarding the received COVID-19 vaccinations. The secondary objectives were to identify the potential factors of COVID-19 vaccine side effects and their correlates.

## Methods

### Study design and settings

We carried out a comparative cross-sectional study in Tanta University Hospitals (TUHs) among HCWs who had been vaccinated with either the first or the second dose. The study was conducted from November 20th, 2021, to January 20th, 2022. Tanta University Hospitals are the largest medical facility located in Egypt’s middle of the Nile Delta. Approximately 5100 nurses and technicians and 2800 physicians are working in TUHs.

### Participants

The inclusion criteria for this study were the HCWs (physicians, nurses, technicians, and others) in Tanta University Hospitals who have been vaccinated with the COVID-19 vaccine since the vaccine’s approval in Egypt. The HCWs who received the *Oxford -AstraZeneca*, *Sinopharm*, and *Sinovac* vaccines were included if they received either the first or second dose from March 2021 until January 2022 by the time of filling out the questionnaire. Nearly 95% of them were vaccinated with either AstraZeneca, Sinopharm, or Sinovac vaccines by the time of this study. We excluded only healthcare workers who did not receive the vaccine during the study period. The sample size was calculated using Epi Info 7 software. The criteria of sample size calculation were based on 95% confidence limit, the prevalence of complications at 85%, as expressed by three experts working in the vaccination center of TUHs, with a margin of error of 3% and a design effect of two. The calculated sample size was found to be 1088. We recruited 1246 participants to compensate for any incomplete questionnaires. We selected study participants by systematic sampling.

### Study tool

A structured self-administered questionnaire, adapted from Kim et al. study in China [24]. The questionnaire was reviewed and pilot tested on 30 HCWs to check the acceptability and clarity of the questions. The pilot responses were not included in the final analysis. We calculated Cronbach’s alpha for internal consistency and found it at 0.711. A panel of seven experts tested content and face validity. The experts were asked independently to review each item using 4 points ordinal scale (one = disagree, two = need modification, three = agree, four = highly agree). The content validity index was estimated. To obtain the content validity index at the item level, the number of experts judging the item as relevant or clear (rating 3 or 4) was divided by the total number of experts. The item will be suitable if it exceeds 0.79.

The questionnaire included four main sections addressing the following data:

*Section I* includes demographic data such as age, sex, occupation, smoking status, and smoking duration, if present. With three questions asking about the received vaccine and the presence of chronic diseases,

*Section II* This section aimed to assess the adverse effects shortly after receiving the COVID-19 vaccine (within 72 h). The healthcare worker was able to choose from 23 questions enumerating adverse effects that WHO and other studies have previously documented following vaccination, with the possibility to add any non-mentioned side effects and a question asking about the duration of the experienced side effects. For the occurrence of each side effect, the respondents answered yes or no.

*Section III* included three questions about previous COVID-19 infections, either diagnosed or experienced suspected symptoms without a diagnosis. Participants answered the question by yes or no, and for the question of diagnosis, they mentioned the method of diagnosis.

*Section IV* contained four questions answered by “agree” or “disagree” to measure the perception and knowledge of the vaccines and their opinion on vaccine safety.

Data collection was through direct interviewing of the study participants. The questionnaire was self-administered, and the data collectors were present to fill it in and explain any queries.

### Statistical analysis

The collected data were organized, tabulated, and statistically analyzed using SPSS version 19 (Statistical Package for Social Studies) created by IBM, Illinois, Chicago, USA. The range, mean, and standard deviation were calculated for numerical values. The number and percentage were calculated for categorical variables, and the Chi-squared test tested differences between subcategories. If in 20% or more of the cells, the number of expected counts is less than five, Fisher’s exact test was used instead. The level of significance was adopted at *p* < 0.05.

## Results

The total number of studied healthcare workers was 1246. Participants who received the AstraZeneca vaccine were relatively younger (34.4 ± 11.45) than their peers receiving other vaccines (37.8 ± 11.83 and 35.0 ± 11.31). The highest frequency of the age group 20–30 received AstraZeneca (45.5%). Meanwhile, among other age categories, the highest frequency received Sinopharm. The distribution of age groups in relation to the type of vaccines was significantly different. Concerning sex, the highest frequency of males (52.7%) received AstraZeneca, and differences in the type of vaccination in relation to sex were statistically significant. The distribution of participants in relation to the type of vaccination and job was statistically significant, with the highest frequency of physicians (46.6%) who received AstraZeneca. The highest frequency of smokers received Sinopharm (66.7%). Those receiving two doses of AstraZeneca were 39.6%, significantly higher than 36.0% and 24.4% for the two other vaccines. The presence of chronic diseases did not significantly affect the type of vaccine received. More than one-fourth (26.8%) reported a previously confirmed COVID-19 infection. However, there are no significant differences in the type of vaccination in relation to previous experience with COVID-19 infections (Table 1).

**Table 1:** Distribution of studied health care workers by type of vaccine

Variables	AstraZeneca		Sinopharm		Sinovac		Total		*χ*^2^	*p*
	*n*	%	*n*	%	*n*	%	*n*	%		
Age in years										
20	258	45.4	154	27.1	156	27.5	568	100	72.624	< 0.001
30	39	18.2	111	51.9	64	29.9	214	100		
40	86	34.4	94	37.6	70	28.0	250	100		
50	62	31.6	94	48.0	40	20.4	196	100		
60	5	27.8	8	44.4	5	27.8	18	100		
Range	20–63		20–63		20–63		20–63			
Mean ± SD	34.4 ± 11.45		37.8 ± 11.83		35.0 ± 11.31		35.80 ± 11.65			
Sex										
Males	147	52.7	76	27.2	56	20.1	279	100	42.800	< 0.001
Females	303	31.3	385	39.8	279	28.9	967	100		
Job										
Physicians	271	46.6	163	28.1	147	25.3	581	100	65.378	< 0.001
Nurses	146	26.0	256	45.6	160	28.5	562	100		
Technicians	24	34.3	32	45.7	14	20.0	70	100		
Others	9	27.3	10	30.3	14	42.4	33	100		
Smoking										
Non-smokers	444	36.7	437	36.1	329	27.2	1210	100	14.142	0.001
Smokers	6	16.7	24	66.7	6	16.7	36	100		
Doses										
One	103	27.9	145	39.3	121	32.8	369	100	14.314	< 0.001
Two	347	39.6	316	36.0	214	24.4	877	100		
Diabetes										
Negative	439	36.3	446	36.9	323	26.7	1208	100	0.944	0.624
Positive	11	28.9	15	39.5	12	31.6	38	100		
Hypertension										
Negative	435	36.5	437	36.7	320	26.8	1192	100	1.949	0.377
Positive	15	27.8	24	44.4	15	27.8	54	100		
Allergy										
Negative	440	36.3	448	37.0	324	26.7	1212	100	0.838	0.658
Positive	10	29.4	13	38.2	11	32.4	34	100		
Previous COVID-19 infection										
None	295	37.0	288	36.1	215	26.9	798	100		
Confirmed	155	34.6	173	38.6	120	26.8	448	100	0.943	0.624
Total	450	36.1	461	37.0	335	26.7	1246	100		

Nearly two-fifths of participants (65.4%) believed in vaccine safety. Significantly, more participants (46.9%) who received AstraZeneca were concerned about thrombotic complications. Trust in governmental policy for vaccinations and the necessity to get the vaccine were reported by 55.5% and 63.6% (Table 2).

**Table 2:** Perceptions regarding the received vaccines among the participants

Variables	AstraZeneca (*n* = 450)		Sinopharm (*n* = 461)		Sinovac (*n* = 335)		Total (*n* = 1246)		*χ*^2^	*p*
	*n*	%	*n*	%	*n*	%	*n*	%		
Believe in the vaccine’s safety	279	66.0	301	65.3	217	64.8	815	65.4	0.132	0.936
Concerned about thrombotic complications	211	46.9	163	35.4	114	34.0	488	39.2	17.780	< 0.001
Trust governmental policy for vaccination	256	56.9	258	56.0	178	53.1	692	55.5	1.150	0.563
Vaccine is necessary	312	69.3	302	65.5	178	53.1	792	63.6	22.955	< 0.001

**Fig. 1:**
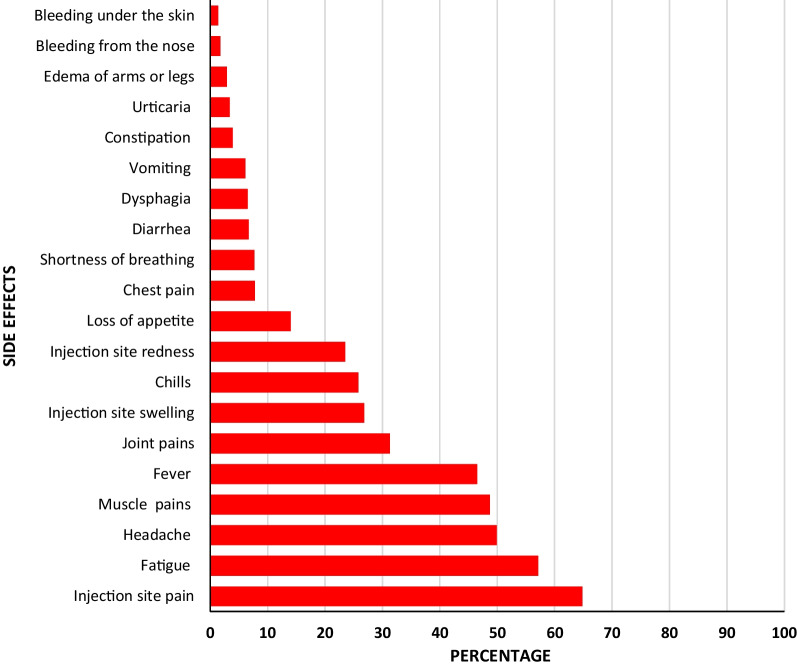
Distribution of studied participants by the prevalence of side effects

The most frequently reported side effects were injection site pain (65.4%), fatigue (57.6%), headache (56.3%), and fever (45.1%). Nearly one-fourth (23.4%) of participants reported that side effects affected their working capacity. The majority (95.9%) reported the duration of side effects as being less than 2 weeks. Most side effects tend to be more frequently reported among participants receiving AstraZeneca than the other two vaccines (Table 3 and Fig. 1). Sex differences in relation to vaccine side effects were not significant (Table 4). Again, side effects in relation to socio-demographic factors and the presence of chronic illness were not also found to be significant (Table 5).

**Table 3:** Self-reported side effects by type of vaccines

Side effects	AstraZeneca (*n* = 450)		Sinopharm (*n* = 461)		Sinovac (*n* = 335)		Total (*n* = 1246)		*χ*^2^	*p*
	*n*	%	*n*	%	*n*	%	*n*	%		
Fever	336	74.7	128	27.8	116	34.6	580	46.5	227.50	< 0.001
Vomiting	35	7.8	29	6.3	12	3.6	76	6.1	5.949	0.051
Diarrhea	41	9.1	22	4.8	20	6.0	83	6.7	7.247	0.027
Headache	286	63.6	207	44.9	129	38.5	622	49.9	55.056	< 0.001
Fatigue	334	74.2	220	47.7	157	46.9	711	57.1	84.712	< 0.001
Chills	165	36.7	92	20.0	65	19.4	322	25.8	43.093	< 0.001
Muscle pains	285	63.6	173	37.5	148	44.2	607	48.7	65.525	< 0.001
Joint pains	192	42.7	97	21.0	101	30.1	390	31.3	49.807	< 0.001
Urticaria	19	4.2	20	4.3	3	0.9	42	3.4	8.628	0.013
Shortness of breathing	52	11.6	27	5.9	17	5.1	96	7.7	14.857	0.001
Chest pain	44	9.8	33	7.2	20	6.0	97	7.8	4.278	0.118
Edema of arms or legs	12	2.7	15	3.3	9	2.7	36	2.9	0.347	0.841
Loss of appetite	102	22.7	42	9.1	30	9.0	174	14.0	44.400	< 0.001
Dysphagia	51	11.3	21	4.6	9	2.7	81	6.5	28.179	< 0.001
Constipation	12	2.7	24	5.2	12	3.6	48	3.9	4.055	0.132
Bleeding under the skin	3	0.7	9	2.0	6	1.8	18	1.4	3.030	0.220
Bleeding from the nose	14	3.1	3	0.7	6	1.8	23	1.8	7.616	0.022
Injection site										
Pain	336	74.7	254	55.1	217	64.8	807	64.8	38.215	< 0.001
Redness	164	36.4	80	17.4	49	14.6	293	23.5	66.271	< 0.001
Swelling	183	40.7	83	18.0	68	20.3	334	26.8	69.496	< 0.001
Side effects affect work capacity	151	33.6	71	15.4	70	20.9	292	23.4	43.474	< 0.001
Duration of symptoms										
< 2 weeks	422	93.8	444	96.3	329	98.2	1195	95.9	12.010	0.017
2–4 weeks	22	4.9	11	2.4	3	0.9	36	2.9		
4 weeks	6	1.3	6	1.3	3	0.9	15	1.2

**Table 4:** Self-reported side effects by sex

Side effects	Males (*n* = 279)		Females (*n* = 967)		*χ*^2^	*p*
	*n*	%	*n*	%		
Systemic side effects						
Fever	160	57.3	420	43.4	16.849	< 0.001
Vomiting	19	6.8	57	5.9	0.317	0.573
Diarrhea	25	9.0	58	6.0	3.057	0.080
Headache	132	47.3	490	50.7	0.978	0.323
Fatigue	171	61.3	540	55.8	2.623	0.105
Chills	107	38.4	215	22.2	29.351	< 0.001
Muscle pains	158	56.6	449	46.4	9.014	0.003
Joint pains	92	33.0	298	30.8	0.469	0.493
Urticaria	8	2.9	34	3.5	0.280	0.597
Shortness of breathing	21	7.5	75	7.8	0.016	0.899
Chest pain	20	7.2	77	8.0	0.190	0.663
Edema of arms or legs	6	2.2	30	3.1	0.699	0.403
Loss of appetite	55	19.7	119	12.3	9.888	0.002
Dysphagia	31	11.1	50	5.2	12.571	< 0.001
Constipation	12	4.3	36	3.7	0.195	0.658
Bleeding under the skin	3	1.1	15	1.6	FET	0.591
Bleeding from the nose	12	4.3	11	1.1	11.960	0.001
Injection site						
Pain	175	62.7	632	65.4	0.658	0.417
Redness	64	22.9	229	23.7	0.066	0.797
Swelling	71	25.4	263	27.2	0.338	0.561

*FET* Fisher’s Exact test

**Table 5:** Potential factors associated with COVID-19 vaccine side effects

Variables	Side effect of COVID-19 vaccines				*χ*^2^	*p*
	No side effects		Side effect			
	*n*	%	*n*	%		
Age in years						
20	49	8.6	519	91.4	6.968	0.138
30	18	8.4	196	91.6		
40	16	6.4	234	93.6		
50	25	12.8	171	87.2		
60	3	16.7	15	83.3		
Sex						
Males	18	6.5	261	93.5	2.674	0.102
Females	93	9.6	874	90.4		
Job						
Physicians	51	8.8	530	91.2	4.372	0.224
Nurses	46	8.2	516	91.8		
Technicians	11	15.7	59	84.3		
Others	3	9.1	30	90.9		
Smoking						
Non-smokers	109	9.0	1101	91.0	FE	0.765
Smokers	2	5.6	34	94.4		
Doses						
One	39	10.6	330	98.4	1.782	0.182
Two	72	8.2	805	91.8		
Diabetes						
Negative	107	8.9	1101	91.1	FE	0.769
Positive	4	10.5	34	89.5		
Hypertension						
Negative	106	8.9	1086	91.1	FE	0.810
Positive	5	9.3	49	90.7		
Allergy						
Negative	109	9.0	1103	91.0	FE	0.762
Positive	2	5.9	32	94.1		
Total	111	8.9	1135	91.1		

Fisher’s exact test

## Discussion

This survey-based study aimed to assess the perceptions and frequency of self-reported side effects of Tanta University Hospitals’ HCWs about COVID-19 vaccines and identify their associated factors. The vaccine’s side effects could be classified as either local or systemic, with severity ranging from mild to moderate. Regardless of the experienced side effects, most HCWs vaccine recipients accepted the challenge of stopping the fatal pandemic [25, 26]. The study participants accepted the vaccination due to their high degree of health literacy and scientific enthusiasm. Also, the expected benefits influenced respondents’ decisions more than the expected adverse effects.

Many of the participant HCWs were young and females, which was similarly reported by Elgendy et al. Among the general Egyptian population, they stated that most of the participants were women and younger (< 40 years old), as they were more interested in taking part in the survey and sharing their experiences than the other groups. This could also be because young people and women are more susceptible to post-vaccine adverse effects than seniors and males [22].

In our study, nearly half (48%) of the over-50-year-old participants received the inactivated Sinopharm vaccine. This is evidenced by a study of the efficacy and safety in older age groups, which concluded that inactivated COVID-19 vaccinations revealed promising antibody responses with fewer side effects [27]. The current study showed that the highest percentage of viral vector-based AstraZeneca vaccine uptake was among the youngest age group. This comes in accordance with Dziedzic et al. study, as most recipients of AstraZeneca vaccine were in the younger age group than in the older one [28].

The highest percentage (52.7%) of male participants received AstraZeneca vaccine in this study, while the highest percentage (39.8%) of females received Sinopharm vaccine; this may be attributed to some reports of the occurrence of potential thrombotic episodes in females [29]. However, the World Health Organization’s and European Medicines Agency’s (EMA) assured no indication that Oxford–AstraZeneca vaccine was associated with thromboembolic events [30, 31]. The issue of potential thromboembolic events to AstraZeneca vaccine is challenging to decide the causality from a coincidence since the COVID-19 infection is also linked with blood clotting [32, 33].

The current study revealed that the inactivated Sinopharm vaccine was the most frequently used type among HCWs with chronic morbidities such as diabetes, hypertension, and allergies rather than the viral vector-based AstraZeneca vaccine as recommended by the Egyptian MOH regarding the selection of the type of vaccine according to the medical history of the recipient taken before vaccination. These findings are consistent with WHO recommendations for the safety and effectiveness of inactivated vaccines for the vaccination of individuals having comorbidities or underlying diseases that put them at risk of developing severe COVID-19 disease [34].

In our study, most participants believed in the vaccine’s safety and confirmed its necessity because of their scientific and medical backgrounds. This was in agreement with Raude et al. who stated that HCWs have a favorable attitude regarding COVID-19 vaccines [35]. Because of their continuous interaction with COVID-19 patients and the need to protect themselves from the risk of infection, HCWs have a positive attitude towards vaccination. According to the health belief model (HBM), which includes belief in the efficacy, perceived benefits, and perceived vulnerability to COVID-19, which is used to drive vaccination, these findings emphasize the importance of spreading clear, accurate, and comprehensive information by every possible means, like mass media and health education campaigns to motivate vaccination. This positive attitude of Egyptian HCWs appears to be consistent with the attitude of medical professionals in the UK and Poland, as the majority of respondents agreed that vaccination against COVID-19 is highly recommended and proved pro-vaccine behaviors, as seen in other countries. In addition, the findings of a survey conducted by Babicki et al. revealed that persons with a higher level of education and healthcare personnel have a more favorable attitude towards the COVID-19 vaccination [36–40].

The present study’s most frequently reported side effects were injection site pain, fatigue, headache, fever, joint pain, and injection site swelling. Similar findings were reported by various published studies, which reported that the most common post-vaccination side effects were pain, swelling at the vaccine injection site, fatigue, muscle, and joint pain, lethargy, dizziness, fever, and headache [22, 41].

The most commonly reported side effect among the studied HCWs who received the three types of vaccines was fatigue. Many Egyptian, large-scale Arab world and global studies concluded that fatigue was one of the most prevalent s observed following COVID-19 vaccination [4, 22, 28, 42–44].

Our study showed that HCWs who got the viral vector AstraZeneca vaccine were more likely to experience post-vaccination systemic and local side effects that influenced the work capacity in one-third of participants compared to those who received Sinopharm and Sinovac vaccines. This was in line with Zahid’s study in Bahrain, which revealed that AstraZeneca had more side effects when compared to Sinopharm vaccine [42]. Among participants who received Oxford–AstraZeneca vaccine, the most overall self-reported side effects were fever, injection site pain, and fatigue, which were similarly reported among Saudi HCWs [43].

Also, the most frequent side effects among participants who received Sinopharm were pain in the injection site and fatigue, which is consistent with other studies conducted to report the adverse effects of Sinopharm vaccine [22, 45].

Pain at the injection site was the most common local side effect experienced by participants who received the Sinovac vaccine, similarly reported by a Turkish study among nurses vaccinated with Sinovac [46]. To alleviate this very common adverse effect, it is recommended to lower the patient’s arm to be injected to reduce pain. An injection into a relaxed muscle causes minor discomfort compared to injection into a tense one. Vaccines should also be stored at a low temperature; the Sinopharm COVID-19 vaccine should be stored at a normal refrigerator temperature. Injections without adequate warming may increase the likelihood of pain at the injection site [18, 43].

Most participants’ side effects were temporary and lasted from days to less than 2 weeks. This finding supports the fact that the majority of the post-COVID-19 vaccination adverse effects are self-limiting, and the recipients recover promptly; none of the symptoms is severe enough to necessitate hospitalization. According to the vaccine recipients in many studies, the post-vaccine symptoms are usually minimal. The symptoms were minor, insignificant, and did not threaten their lives [22, 41, 47].

The highest frequency of vaccine-related side effects was reported among the age group from 40 to less than 50 years. This is in coherence with an Iraqi study that revealed that middle-aged HCWs were more at risk of adverse vaccination effects [48].

Concerning gender-based discrepancy of the vaccines’ side effects, male participants reported higher frequencies of side effects, especially the systemic ones, than females. These findings are in line with the results of a similar study among Polish HCWs, which reported that male participants had a greater rate of common mild systemic adverse events [28]. In contrast, female participants experienced more frequent local adverse effects. Interestingly, Italian female HCWs had a more robust immunological response to the vaccine and higher serological markers following COVID-19 vaccination, implying a link with more frequent post-vaccination self-reported side effects [49]. Besides, selection and information bias may also play a role in developing gender-based discrepancies. Hence, gender-adjusted analyses are required when investigating the self-reported outcomes of COVID-19 vaccines [50]. The smoker participants had a higher percentage of post-vaccination side effects than the non-smoker HCWs. The same was found by Mohsin et al., who stated that smokers were 3.6 times significantly higher than non-smokers to report side effects [51].

In the current study, HCWs reported a higher percentage of side effects than the second vaccination dose following the first dose. Like the findings of Khadka et al. a study in which participants experienced no more side effects following the second dose of vaccination than after the first [41].

In our study, HCWs with comorbid chronic conditions and allergies surprisingly demonstrated lower frequencies of COVID-19 vaccine-related side effects. This may be attributed to the fact that most of them received the safer inactivated vaccines. Chronic health problems were linked to a higher frequency of local and systemic vaccination-related side events. However, no definitive link has been shown between chronic diseases and the occurrence of post-vaccination side effects. It has been proposed that underlying or undetected health issues could influence adverse reactions to vaccination.

On the contrary, a weakened immune response due to comorbid disorders can be associated with decreased, attenuated immunological responsiveness, resulting in fewer adverse outcomes. In our study, HCWs with comorbid chronic conditions and allergies surprisingly demonstrated lower frequencies of COVID-19 vaccine-related side effects. This may be attributed to the fact that most of them received the safer inactivated vaccines. Chronic health problems were linked to a higher frequency of local and systemic vaccination-related side events. However, no definitive link has been shown between chronic diseases and the occurrence of post-vaccination side effects. It has been proposed that underlying or undetected health issues could influence adverse reactions to vaccination. On the contrary, a weakened immune response due to comorbid disorders can be associated with decreased, attenuated immunological responsiveness, resulting in fewer adverse outcomes [28, 52, 53].

The study revealed that nearly all the side effects that occurred were similar to those reported in the literature, indicating that most COVID-19 vaccines` side effects are almost known at this time. The majority of them are non-life threatening, with most cases being mild to moderate in intensity and resolving in a few days. Good knowledge of vaccinations and their adverse effects was substantially related to the vaccination acceptance rate [44]. As a result, contradicting the rumors, misconceptions, and conspiracy theories regarding COVID-19 vaccinations and their real adverse effects could boost public trust and confidence in COVID-19 vaccines. Based on the results of the study, the following points are recommended:

1. Academic institutions should conduct further independent (non-sponsored) epidemiological research on the adverse effects of all available authorized COVID-19 vaccines, especially mRNA vaccines, which were not included in this study and after receiving the booster doses.
2. More independent community-based studies on vaccination safety are urgently needed to better understand the potential risk factors for vaccine side effects, boost public trust in vaccines, and accelerate their uptake.
3. As the vaccination campaign continues, there is a need to monitor additional reports on vaccines’ short-term and long-term side effects.

One of the strengths of this study is that it is one of the earliest studies to investigate the COVID-19 vaccines’ side effects among Egyptian HCWs. Obtaining sufficient and correct information about vaccines is essential to boost vaccination uptake. The current study provides authorities with in-depth insights into the anticipated challenges and issues. The first limitation of this study is that it was a single center using convenience sampling, which may restrict the generalizability of the findings. However, the large sample size could help increase the validity of the results. In addition, the use of self-reported data as the study investigators did not verify the receipt of vaccination doses by the participants, nor did they document their claimed symptoms, which are subjective and may involve recall bias. In addition, the vaccine’s latent effects were not investigated or included in this study.

## Conclusion

Overall, HCWs attitudes toward the national vaccination program were favorable. The most important conclusion is that with all three vaccines received by HCWs in this study—namely AstraZeneca, Sinopharm, and Sinovac—the first and second dose post-vaccination side effects were minor and predictable, mainly in the form of injection site pain, fatigue, headache, muscle pains, fever, joint pain, and injection site swelling. The study demonstrated that COVID-19 vaccinations approved in Egypt have a mainly safe profile, with mild and self-resolving side effects. The reported primarily non-life-threatening short-term adverse effects may contradict conspiracy theories and encourage vaccine-apprehensive public members about the vaccine’s safety.

## Data Availability

Data are available upon reasonable request from the corresponding author.

## Abbreviations

EMA: European Medicines Agency
HCWs: Health care workers
TUHs: Tanta University Hospitals
WHO: World Health Organization
