# Correction: Perceptions and experiences of COVID-19 vaccines’ side effects among healthcare workers at an Egyptian University Hospital: a cross-sectional study

**DOI:** 10.1186/s41182-022-00476-7

**Published:** 2022-11-02

**Authors:** Hisham Ahmed Orebi, Hesham Elsayed Emara, Abdallah Ahmoud Alhindi, Mohamed Reda Shahin, Arwa Hassan Hegazy, Ibrahim Ali Kabbash, Shimaa M. Saied

**Affiliations:** 1 Faculty of Medicine, Tanta University, Tanta, Egypt; 2 Public Health and Community Medicine Department, Faculty of Medicine, Tanta University, Tanta, Egypt

## Correction to: Tropical Medicine and Health (2022) 50:37 https://doi.org/10.1186/s41182-022-00427-2

Following publication of the original article [1], the authors reported an error in ethical approval number. The ethical review number should be corrected from 34474/2/2021 to 35033/11/21.

The correct version has been provided below.

**Ethics approval and consent to participate**

The Ethical Committee of Scientifc Research in the Tanta Faculty of Medicine approved the research before starting the study (No. 35033/11/21). For participants’ consent, we inserted a written consent in the introductory page of the questionnaire, and all participants gave informed consent before answering the questions. Participation in this study was voluntary.

The original article [1] has been corrected.
